# Uterine Ectopic Pregnancies and Live Births: Systematic Review of the Literature and Concepts Underlying Favorable Outcomes

**DOI:** 10.3390/medicina61111915

**Published:** 2025-10-25

**Authors:** Guglielmo Stabile, Laura Vona, Stefania Carlucci, Anna Pitsillidi, Stefano Restaino, Giuseppe Vizzielli, Luigi Nappi

**Affiliations:** 1 Department of Medical and Surgical Sciences, Institute of Obstetrics and Gynaecology, University of Foggia, 71122 Foggia, Italy; guglielmost@gmail.com (G.S.); s.carlucci86@gmail.com (S.C.);; 2 Department of Obstetrics and Gynaecology, Rheinland Klinikum Neuss, Preußenstrasse 84, 41464 Neuss, Germany; anna.pitsillidi@gmail.com; 3 Department of Maternal and Child Health, Obstetrics and Gynecology Clinic, University-Hospital of Udine, 33100 Udine, Italy; stefano.restaino@asufc.sanita.fvg.it (S.R.); giuseppevizzielli@yahoo.it (G.V.); 4 Clinic of Obstetrics and Gynecology, “S. Maria della Misericordia” University Hospital, Azienda Sanitaria Universitaria Friuli Centrale (ASUFC), 33100 Udine, Italy

**Keywords:** ectopic pregnancy, intramural pregnancy, interstitial pregnancy, cervical pregnancy, and live birth

## Abstract

*Background and Objectives*: Uterine ectopic pregnancy includes uterine extraendometrial forms such as cervical, intramural, and interstitial pregnancies, whose incidence is increasing with prior uterine surgery and assisted reproduction. Unlike cesarean scar pregnancy, which is known to occasionally progress to term, the potential for these other types to continue beyond the first trimester remains poorly defined. This review evaluates reported cases carried to viability, focusing on maternal and neonatal outcomes and identifying prognostic factors influencing progression. *Materials and Methods*: This systematic review was conducted in accordance with PRISMA guidelines and registered in PROSPERO (CRD420251070864). Comprehensive searches of PubMed, Scopus, and Web of Science up to June 2025 identified English-language case reports of uterine ectopic pregnancies (cervical, intramural, or interstitial) resulting in live birth. Data on maternal characteristics, clinical presentation, pregnancy course, delivery outcomes, and neonatal parameters were extracted. Study quality was assessed using the Joanna Briggs Institute checklist for case reports. *Results*: Uterine ectopic pregnancies were frequently misdiagnosed, with definitive diagnosis established only at delivery in 85% of cases. The majority of patients presented with abdominal pain or vaginal bleeding, and maternal morbidity was considerable: two-thirds required hysterectomy, and blood transfusions were often necessary due to severe hemorrhage. Fourteen live births were reported (nine interstitial, four cervical, and one intramural). Neonatal survival was primarily dependent on gestational age at delivery, while successful continuation of pregnancy appeared favored by implantation in more distensible myometrial regions and the presence of residual endometrial tissue. *Conclusions*: An increased amount of endometrium and greater myometrial distensibility at the implantation site enhance the likelihood of uterine ectopic pregnancies progressing to viability. These factors should guide early diagnosis, patient counseling, and individualized management, considering gestational age, implantation type, and future fertility goals.

## 1. Introduction

Ectopic pregnancy is defined as the implantation of a fertilized ovum outside the endometrial cavity.

Traditionally, ectopic pregnancy has been equated with extrauterine pregnancy, predominantly referring to tubal implantation. However, no distinct anatomical barriers exist between the uterine cavity, fallopian tubes, and peritoneal cavity [1]. Consequently, implantation can occur at any point along the continuum from the ovary to the cervical canal [2].

The term normal uterine pregnancy lacks specificity, as it does not clearly distinguish between a gestation implanted within the endometrial cavity and a uterine ectopic pregnancy located within the uterus but outside the endometrial lining. Furthermore, these pregnancies often present with mild clinical symptoms and may contain a live embryo or fetus, complicating differentiation from normally sited intrauterine pregnancies [3].

The rising prevalence of uterine surgical procedures—such as cesarean section, myomectomy, and dilation and curettage—that compromise endometrial integrity, together with the increasing incidence of sexually transmitted diseases and the expanding use of assisted reproductive technologies, has contributed to the growing incidence of abnormal implantation [4,5]. Notably, there has been a significant increase in non-tubal uterine ectopic pregnancies, including cesarean scar, cervical, intramural, and interstitial types [6]. Although these forms are relatively rare, they pose substantial diagnostic challenges due to their intrauterine location and extra-endometrial implantation.

Improving the ultrasonographic diagnosis of uterine ectopic pregnancy requires first establishing a standardized definition of a normally implanted intrauterine pregnancy. Subsequently, consensus on diagnostic criteria is essential to accurately distinguish between different forms of ectopic implantation [1]. Current guidelines emphasize that each ectopic pregnancy type can be characterized by specific anatomical landmarks, which are critical for achieving an accurate diagnosis and management [7].

This is particularly important in cases of uterine ectopic pregnancies, defined by evidence of trophoblast invasion beyond the endometrial–myometrial junction, but not outside the uterine visceral/broad ligament peritoneum. In these cases, both the exact position of the gestational sac within the uterine cavity and the degree of myometrial involvement are critical [1]. These factors help guide the decision between conservative and surgical management options, enabling individualized care based on the specific anatomical and clinical context.

Cervical pregnancies (CEPs) are a rare form of uterine ectopic pregnancy, accounting for approximately 1% of all ectopic pregnancies [8]. Risk factors for CEPs are previous operations of the uterus, medically assisted reproduction, and Asherman’s syndrome [9,10]. Cervical ectopic pregnancy is characterized by implantation of the gestational sac within the cervical stroma, below the level of the internal os. Sonographic diagnostic criteria include (1) the presence of a gestational sac below the internal os, (2) absence of the sliding sign, and (3) peritrophoblastic blood flow detected on color Doppler imaging [7]. Any pregnancy implanted in the posterior cervix should be classified as a cervical ectopic pregnancy, irrespective of prior obstetric history. In contrast, anteriorly located gestations in women with a history of cesarean section require differentiation from cesarean scar ectopic pregnancy.

Intramural pregnancy, which accounts for approximately 1% of all ectopic pregnancies, is characterized by implantation within the myometrium above the level of the internal os, distinguishing it from cervical and cesarean scar pregnancies [11]. They most commonly occur following prior uterine surgery, such as myomectomy, uterine perforation, and uterine curettage. In some cases, implantation may occur within a focus of adenomyosis [12]. Intramural pregnancies do not involve the interstitial portion of the fallopian tubes, allowing differentiation from interstitial tubal ectopic pregnancies. Because they may implant anywhere within the uterine corpus, they are often more challenging to diagnose than cervical or lower uterine segment scar pregnancies [13]. The key diagnostic feature is trophoblastic invasion beyond the endometrial–myometrial junction above the internal os [14].

Interstitial pregnancy is a type of ectopic gestation implanted within the intramural portion of the fallopian tube, accounting for less than 3% of ectopic pregnancies [15,16]. Due to its location in the thicker, more compliant segment of the tube near the uterus, interstitial pregnancies have a greater capacity to grow larger and may contain a live embryo or fetus, thereby increasing the risk of severe complications such as uterine rupture [17]. Historically, interstitial pregnancies have been distinguished from more distal isthmic and ampullary ectopic pregnancies because most tend to extend laterally into the proximal tubal segment; however, pregnancies confined solely to the interstitial segment are relatively uncommon [18]. A key diagnostic hallmark is the interstitial line sign, which represents a thin echogenic line of the interstitial portion of the tube adjacent to the medial aspect of the gestational sac and lateral uterine cavity [19]. Additionally, the gestational sac is partially surrounded by myometrium. Early diagnosis remains challenging, and it is critical to differentiate interstitial pregnancies from cornual, angular, and uterine ectopic pregnancies, as these conditions differ significantly in clinical behavior, management, and outcomes [20]. Notably, cornual pregnancy, which develops in the rudimentary horn of a unicornuate uterus outside the main uterine cavity (as reported by ESHRE classification 2020) [1], was excluded from our review, as we focused solely on uterine ectopic pregnancies occurring in anatomically normal uteri.

Scar pregnancy is a rare form of ectopic gestation characterized by implantation of the embryo within a cesarean section scar defect located in the myometrium of the uterine isthmus [21]. Its incidence ranges between 0.04% and 0.05% of all pregnancies [22]. Unlike other ectopic pregnancies, CSP may advance to term or near-term, raising debate over whether pregnancy termination should be the sole management approach [23]. Nonetheless, cesarean scar pregnancies exhibiting detectable embryonic or fetal cardiac activity and managed via conservative observation are associated with elevated maternal morbidity. Bartels et al. reported that expectant management resulted in a 57% neonatal viability rate; however, 63% of these cases necessitated subsequent hysterectomy secondary to placenta accreta spectrum disorders or uterine rupture [24].

Given the high live birth rate associated with cesarean scar pregnancy (CSP) and the established potential for these uterine ectopic pregnancies to progress to term, CSP cases were excluded from our systematic review. The aim of our review is to evaluate the risk factors, characteristics, and factors that allowed the uterine ectopic pregnancy to reach term and the most correct management for the delivery.

## 2. Materials and Methods

### 2.1. Search Strategy

**Figure 1:**
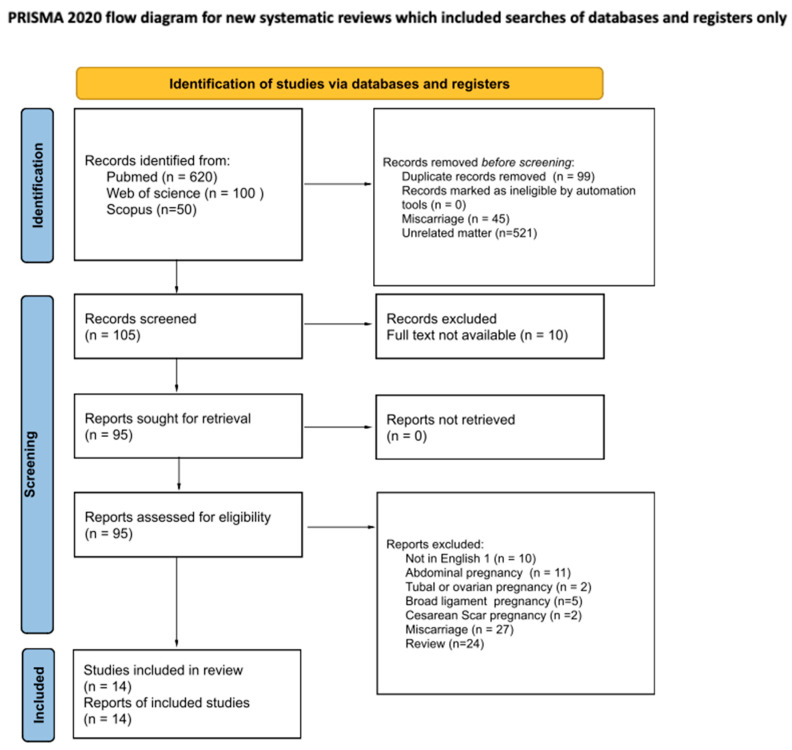
PRISMA flow diagram.

This systematic review was conducted in accordance with the PRISMA guidelines for systematic reviews (see Supplementary Table S1) [25]. Two independent reviewers (L.V. and G.S.) performed a comprehensive literature search of the Web of Science, Scopus, and MEDLINE (PubMed) databases, including all studies published up to June 2025, without any date restrictions. The search strategy combined the following keywords and MeSH terms: ectopic pregnancy, intramural pregnancy, interstitial pregnancy, cervical pregnancy, and live birth. Articles focusing on uterine ectopic pregnancies were considered eligible. The study selection process is detailed in the PRISMA flow diagram (Figure 1).

### 2.2. Eligibility Criteria

Eligible study designs included case reports, randomized controlled trials, prospective controlled studies, prospective cohort studies, retrospective studies, and case series. Only full-text articles published in English were included. Systematic reviews, meta-analyses, letters to the editor, and conference abstracts were excluded. However, reference lists of relevant reviews were manually screened to identify additional eligible studies. Studies with unclear, incomplete, or low-quality data, or those reporting non-quantifiable outcomes, were excluded. Articles not published in English or addressing ectopic pregnancies outside the uterine location were also excluded.

### 2.3. Data Extraction and Risk of Bias Assessment

All records identified through database searches were screened for publication year, citation details, title, authorship, abstract, and full text. Duplicate records were manually identified and removed independently by two reviewers (G.S. and L.V.). Titles and abstracts of the remaining articles were independently screened by the same reviewers to exclude irrelevant studies. Full texts of potentially eligible studies were then independently assessed for inclusion. Discrepancies were resolved by discussion and consensus. The methodological quality of included studies was assessed using the Joanna Briggs Institute (JBI) Critical Appraisal Checklist for Case Reports (Supplementary Table S2). This study has been registered in the PROSPERO database (registration number: CRD420251070864). The inclusion of only case reports in this review presents a risk of bias.

### 2.4. Data Synthesis and Statistical Analysis

Data extracted included patient demographics, obstetric history, gestational age at diagnosis, pregnancy complications, mode of delivery, gestational age at delivery, neonatal weight, and APGAR scores. Postpartum complications and the presence of pathological examination were also recorded. Where possible, continuous variables were reported as means, while discrete and dichotomous variables were presented as percentages. Due to the low number of patients in our review, some data are presented descriptively.

## 3. Results

Our review includes 4 cases of cervical pregnancy, 9 of interstitial pregnancy, and 1 of intramural pregnancy (Table 1). In our analysis, the mean age of patients was 31 years. In the study by Ugwumadu et al. [26], this data was not reported. The average gravity and parity were 1.7 and 0.55, respectively. These data were missing in three studies [27,28,29]. The mean gestational age at diagnosis was 30 + 4 weeks (range 7–38). In 57% of cases, the uterine ectopic pregnancy was mistaken for a normal pregnancy at the ultrasound or RM diagnosis, two of which were cases with placenta previa [29,30], and in two, the uterine body was mistaken for a uterine fibroid [26,31]. In 21% (*n* = 3) of cases, the ectopic pregnancy was mistaken at the US/RM for a cornual pregnancy in a bicornuate uterus. Only in one case [28] was the diagnosis successfully made using MRI, after it had been missed on ultrasound. In Najib’s [32] case, during diagnostic laparoscopy, the interstitial pregnancy was mistaken for a ruptured fibroid, and in the US/RM, it was confused with cervical cancer. In Hill’s case, the laparoscopic examination resulted in the misidentification of an interstitial pregnancy as an abdominal pregnancy [33]. In 85% (*n* = 12) of cases, the diagnosis was made at delivery. Only Scarella et al. suspected an interstitial pregnancy on ultrasound at 20 weeks, with the definitive diagnosis confirmed by MRI at 30 weeks [28]. Köninger et al. [34] diagnosed a cervical pregnancy by ultrasound at 8 weeks.

In 92% of cases, patients presented to the emergency department at least once due to pregnancy-related complications. The most frequent symptoms were abdominal pain, reported in 35.7% (*n* = 5) of cases, followed by vaginal bleeding in 28.5% (*n* = 4) and gestational hypertension in 21.4%. Seventy-five percent of patients presenting with vaginal bleeding were diagnosed with cervical pregnancy. Among these, 100% experienced painless bleeding. In 42.8% (*n* = 6) of cases, signs of fetal distress or anomalies in fetal well-being were observed (oligohydramnios, abnormal CTG findings, various degrees of fetal distress). Breech presentation was reported in 28.5% (*n* = 4) of fetuses.

Cesarean section was performed in 93% (*n* = 13) of cases, and in 30.7% (*n* = 4) of these, an emergency cesarean was required. The mean gestational age at delivery was 33 + 6 weeks. The mean interval between diagnosis and delivery was 2 weeks and 2 days (range: 0–22 weeks).

The average birth weight was 1743 g. The most common APGAR scores were reported at 1 and 5 min, and the median values were 6 and 8, respectively. These data were missing in studies 14 and 11.

In the study by Scarella et al., the neonate born at 28 weeks died 12 h after delivery due to severe respiratory distress caused by early oligohydramnios and pulmonary hypoplasia.

Placenta accreta spectrum (PAS) was diagnosed in 64% (*n* = 9) of cases (in only one-third of these, hysterectomy was not required). Hysterectomy was performed in 64% (*n* = 9) of our patients. In 42.8% (*n* = 6) of cases, at least three units of packed red blood cells were transfused. Histological confirmation was performed in 86% (*n* = 12) of cases.

## 4. Discussion

Uterine ectopic pregnancies are frequently challenging to diagnose and are often misdiagnosed, even when using ultrasound, magnetic resonance imaging (MRI), and, in some cases, laparoscopy. However, Ultrasound and magnetic resonance imaging remain the main methods for diagnosis [1].

The differential diagnosis of uterine ectopic pregnancies, reported in the studies included in our review, includes uterine fibroid, cervical cancer, ruptured fibroid, cornual pregnancy, and abdominal pregnancy. In 85% of cases, the correct diagnosis was made only at the time of delivery. Only Köninger et al. reported a definitive diagnosis of cervical pregnancy during the first trimester [34]. In patients reporting multiple episodes of pain and bleeding during pregnancy, referral to specialized centers for prenatal diagnosis may be beneficial. This approach could facilitate earlier and more accurate diagnosis.

**Figure 2:**
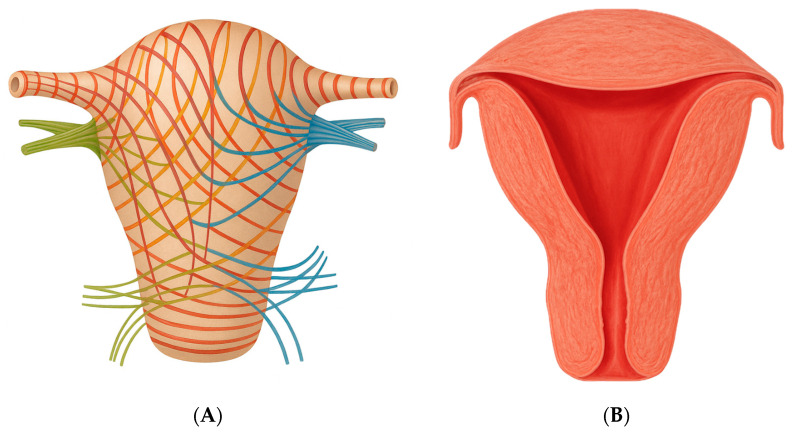
(**A**) The uterine muscular layer and its orientation; (**B**) variation in myometrial thickness across uterine regions [41].

Given that ectopic pregnancy is a recognized risk factor for symptoms such as abdominal pain and abnormal vaginal bleeding, 92% of the patients in our review presented to the emergency department at least once. Specifically, in cases of cervical pregnancy, 3 out of 4 patients presented with painless vaginal bleeding. This is primarily due to the anatomical structure of the cervix, which lacks substantial muscular tissue. Consequently, bleeding tends to be painless yet profuse, as the trophoblastic invasion is not opposed by the contractile capacity needed for effective hemostasis. This contrasts with bleeding in cases of threatened miscarriage in normally implanted pregnancies, where the presence of myometrial musculature aids in limiting blood loss through uterine contractions [Figure 2A,B]. Notably, these contractions, while essential for hemostasis, are also a primary source of the pain typically associated with such events [34]. Interstitial pregnancies are associated with extensive hemorrhage due to the close anatomical relationship between the gestational sac and the intramyometrial arcuate vasculature. This vascular proximity significantly increases the risk of severe maternal morbidity and mortality [39]. Furthermore, the decidua plays a crucial role in regulating trophoblast invasion and spiral artery remodeling. Placenta accreta spectrum (PAS) arises from an underlying endometrial defect and, in ectopic pregnancies—where decidualization is significantly reduced—is characterized by excessive trophoblastic invasion and abnormal vascularization [40]. PAS was diagnosed in 64% of cases, with the spectrum encompassing accreta, increta, and percreta, all associated with a high risk of massive hemorrhage and obstetric complications; in fact, hysterectomy was avoided in only one-third of these cases.

Signs of fetal distress or anomalies in fetal well-being were observed in 42.8% of cases, likely due to abnormal implantation sites, which are associated with placental abnormalities in 64% of cases. In two-thirds of cases, a postpartum hysterectomy was required, leading to permanent loss of fertility. Additionally, transfusions of packed red blood cells were often necessary due to significant intrapartum hemorrhage.

Only one neonate died within 12 h after birth, as a result of prematurity and pregnancy-related complications such as oligohydramnios and pulmonary hypoplasia [28]. Nevertheless, Apgar scores at birth were low in all cases, most likely due to the preterm nature of the deliveries.

It is therefore essential that counseling includes clear information for the patient: while neonatal survival in these cases is comparable to that of normal pregnancies at the same gestational age, the risk of requiring a destructive cesarean section is high, and maternal morbidity and mortality are significantly greater than in cesarean deliveries of uncomplicated pregnancies.

Although the available data in the literature are limited and affected by bias due to the rarity of these cases—which makes clinical management challenging and often results in a very short interval between diagnosis and cesarean section—it can be observed that cervical pregnancies tend to terminate earlier than interstitial pregnancies. This may be partially explained by fetal position in the uterus and the onset of preterm labor triggered by full cervical dilation, which increases the likelihood of spontaneous delivery in cervical pregnancies compared to interstitial ones. 

A total of 28.6% of cases presented in breech position, a higher percentage than typically observed in normal intrauterine pregnancies [42]. This may be due to malposition resulting from the abnormal implantation of the pregnancy, which can prevent the fetus—especially in the third trimester of pregnancy—from assuming a cephalic presentation. Additionally, fetal presentation is dynamic until approximately 32 to 36 weeks, when the majority of fetuses settle into a cephalic position in preparation for delivery; therefore, prematurity itself represents a risk factor for abnormal presentations [43].

The most common ectopic pregnancies are tubal. However, due to anatomical constraints, the fallopian tube has limited capacity and typically ruptures within the early weeks of gestation [1]. In contrast, cesarean scar pregnancies (CSPs), although implanted in an abnormal location, develop within the uterus and can be carried to term in approximately 60% of cases, albeit with a high rate of complications [24].

The authors questioned which factors might allow for the continuation of pregnancy in such a significant proportion of cases. Two main elements appear to play a determining role:

1. The amount of endometrium present at the implantation site, which enables proper development of the syncytiotrophoblast;
2. The ability of the myometrium in that uterine region to expand.

In our study, one case of intramural pregnancy resulted in a live birth [29]. This number increased to four in cases of cervical pregnancies and nine in interstitial pregnancies. Interstitial pregnancy is the most common form of uterine ectopic pregnancy, whereas intramural pregnancy remains extremely rare. This factor may also contribute to the differences in live birth rates observed in our study. Although the implantation mechanism of an intramural ectopic pregnancy and a cesarean scar pregnancy appear to be similar, in the latter, the amount of endometrial tissue available for implantation of the syncytiotrophoblast is generally greater, and the pregnancy tends to grow predominantly within the uterine cavity—unlike in intramural pregnancies.

Furthermore, the myometrium exhibits greater distensibility at the level of the isthmus and fundus, near the interstitial portion of the fallopian tube, which may facilitate the progression of uterine ectopic pregnancies [Figure 2] [44]. It is likely not coincidental that the only reported case of an intramural pregnancy carried to term was located in the uterine fundus—an anatomically more compliant region, similar to the isthmus, capable of accommodating progressive gestational growth. Additionally, in intramural pregnancy, implantation occurs at the site of an adenomyotic focus or within a microscopic sinus tract formed because of uterine trauma, such as previous dilatation and curettage or cesarean scar [29]. This mechanism might underlie the presence of endometrial tissue within the myometrium, which in such cases is minimal. Indeed, intramural pregnancies are rare compared to other types of uterine ectopic pregnancies.

**Figure 3:**
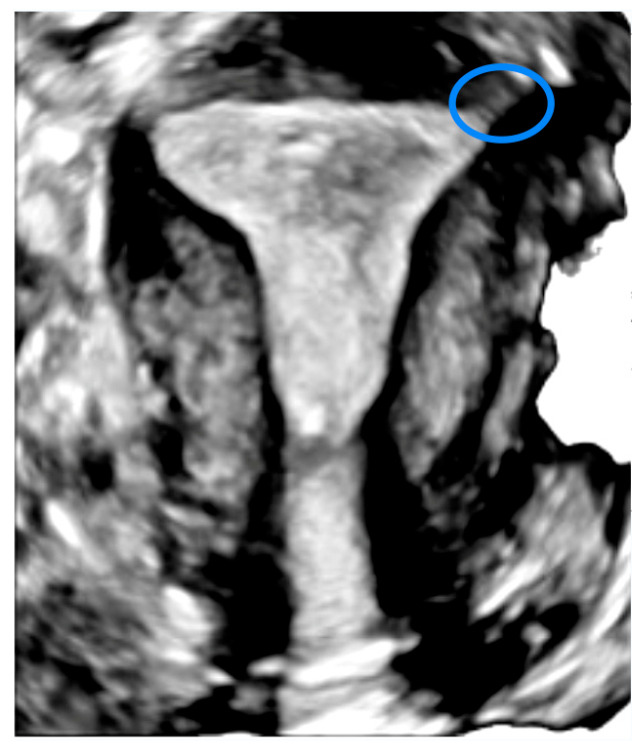
The interstitial segment on a 3D ultrasound image of the uterus—SIEOG, [46].

It is also known that the upper portion of the cervix (about the first centimeter beyond the internal cervical os) contains endometrial tissue [45], which may explain the possibility of implantation and pregnancy development in this area as well. However, the cervix has a far lower capacity for expansion compared to the uterine fundus, due to its limited size. Regarding interstitial pregnancies, the echogenic line extending from the lateral margin of the endometrial cavity through the myometrium toward the uterine serosa corresponds to the endometrium continuing into the interstitial segment of the fallopian tube [Figure 3]. The interstitial line sign, a thin interstitial segment of the Fallopian tube connecting the medial aspect of the gestational sac to the lateral aspect of the uterine cavity, is a key diagnostic feature of interstitial ectopic pregnancies and is most clearly appreciated on 3D imaging, although it can also be identified using conventional 2D ultrasonography [1,39].

## 5. Conclusions

In conclusion, a greater amount of endometrium at the implantation site, along with a more expandable surrounding myometrium, increases the likelihood of successful development of a uterine ectopic pregnancy. These considerations should also be kept in mind during early diagnosis and counseling regarding potential pregnancy termination, considering the gestational age, the type and location of the uterine ectopic pregnancy, as well as the patient’s desire for future fertility. Moreover, the management of these pregnancies at more advanced gestational ages carries a significant risk to the patient’s life.

## Figures and Tables

**Table 1:** Cases in the literature.

Author, Year	Ectopic Pregnancy Type	Cases (*n*)	General Anamnesis Obstetric Anamnesis	GA at the Diagnosis (Weeks)	Complications During the Pregnancy	Delivery Way and Complications	GA at Delivery (Weeks)	Fetal Weight (g) and APGAR	Complications After the Delivery	Pathological Examination
Angela Köninger et al., 2023 [34]	Cervical Pregnancy	1	37 yo ^1^ IIIG IIP 2 CS ^2^	US ^3^ (7 + 3)	Cerclage at 12 weeks Pelvic Pain at 30 weeks	CS UAE ^4^ was planned during the surgery	30	1620 8/8/9	The placenta was left in situ. Hematuria. Re-laparotomy and cervicotomy to evacuate the placenta and repair the bladder injury. Transfusion: 8 Blood units	No
Spryros A. Mesogitis et al., 2001 [35]	Cervical Pregnancy	1	27 yo IG 0P	At delivery (37 + 2)	Vaginal Bleeding at 9 and 33 weeks	Vaginal vacuum extractor	37 + 2	2750 9/10	Excessive Hemorrhage, anuria—Hemorrhagic Shock Total Hysterectomy Transfusion: 4 blood units and 8 platelet units	Yes
H. M. H. Hofmann et al., 1987 [31]	Cervical Pregnancy	1	42 yo IIIG IIP	At delivery (32 + 3)	32 weeks: severe preeclampsia and vaginal bleeding 32 + 3 weeks: Profuse painless bleeding	CS (emergency)	32 + 3	1930 6/7	Placenta Increta Hysterectomy	Yes
I. Cohen et al., 1985 [30]	Cervical Pregnancy	1	36 yo IG 0P	At delivery (26 + 2)	12 weeks: cerclage for severe degree of cervical effacement 25 weeks: Painless vaginal bleeding 26 + 2 weeks: Preterm Premature Rupture of Membranes (pPROM). Cervical cerclage was removed	CS (breech presentation)	26 + 2	1050 9/10	Placenta accreta Hysterectomy Transfusion: 5 blood Units	Yes
Fatemeh Sadat Najib et al., 2021 [32]	Interstitial Pregnancy	1	32 yo IG 0P	At delivery (38)	26 weeks: acute abdomen and hemoperitoneum—interstitial pregnancy was mistaken with a degenerated and bleeding posterior myoma. Blood Transfusion was performed.	CS (breech presentation)	38	2840 8/9	Placenta increta. Hysterectomy	Yes
Shiho Nagayama et al., 2020 [16]	Interstitial Pregnancy	1	41 yo IVG IP	At delivery (28 + 1)	11 weeks: subchorionic hematoma 26 + 6 weeks: early onset preeclampsia 27 + 2 weeks: preeclampsia; lung edema; normal umbilical Doppler; suspect of PAS ^5^. 28 + 1 weeks: severe headache and PA 180/100mmHg	CS (emergency)	28 + 1	926 (−1.4 SD) 3/6	Placenta accreta Hysterectomy 10 blood units and 8 plasma units	Yes
Aiko Kakigano et al., 2018 [27]	Interstitial Pregnancy	1	33 yo Multiparous CS in anamnesis	At delivery (38)	NA	CS	38	3148 NA	Placenta accreta Supracervical hysterectomy	Yes
Yusuke Tanaka et al., 2014 [36]	Interstitial Pregnancy	1	35 yo IG 0P	At delivery (32)	31 weeks fetal growth restriction; umbilical artery Doppler showed reversed end-diastolic velocity.	CS (Breech presentation)	32	1038 (−3.0 SD) 7/9	Placenta accreta—left in situ On day 6 postoperative: fever On day 8, placenta was spontaneously removed	No
Alexandria J. Hill et al., 2013 [33]	Interstitial Pregnancy	1	27 yo IIG IP	At delivery (32)	25 weeks: gestational diabetes 28 weeks: high blood pressure, no preeclampsia 30 weeks: either persistent or intermittent absent end diastolic flow; no stress tests remained reactive until delivery at 32 weeks.	CS (Breech presentation) Before the laparotomy, cystoscopy, and bilateral urethral stents were placed.	32 W	1430 4/4/7	The right tube and ovary were removed with the sac and placenta	Yes
Anibal Scarella et al., 2012 [28]	Interstitial Pregnancy	1	30 yo Multiparous	US (20) DD ^6^ between cornual ectopic pregnancy with placenta accreta and interstitial pregnancy. MRI ^7^ (26 + 1) definitive diagnosis	20 weeks: pPROM; oligohydramnios, placenta increta 25 weeks: pulmonary hypoplasia was diagnosed. 27 + 5 weeks: abdominal discomfort; vaginal bleeding.	CS	28	1000 NA/9 Severe respiratory distress syndrome; death after 12 h	Hysterectomy	Yes
P.H. Ng et al., 2007 [37]	Interstitial Pregnancy	1	27 yo IG 0P	At delivery (38)	NA	CS (Breech presentation)	38	NA Healthy newborn	Adherent Placenta—left in situ. Weekly IM MTX. Placenta was delivered 17 days postoperative	No
Idama T.O. et al., 1998 [38]	Interstitial Pregnancy	1	26 yo IG 0P	At delivery (30)	30 weeks: intermittent abdominal pain; vomiting; Oligohydramnios; tense and tender abdomen.	CS (emergency)	30	1682 1/6	Hemoperitoneum (1200 mL) 3 blood Units	Yes
A.H.N. Ugwumadu et al., 1997 [26]	Interstitial Pregnancy	1	IG 0P	At delivery (33)	During the pregnancy: Abdominal pain 33 weeks: shock; tense and tender abdomen CTG: sinusoidal fetal heart pattern.	CS (emergency)	33	2100 2/7/9	Hemoperitoneum (2000 mL) Placenta accreta Hysterectomy	Yes
Laurent Petit et al., 2012 [29]	Intramural pregnancy	1	36 yo	At delivery (37)	13 weeks: diagnosis of anterior placenta previa	CS	37	NA Healthy newborn	Excessive hemorrhage Hysterectomy 10 blood units and 8 plasma units	Yes

^1^ Years old; ^2^ cesarean section; ^3^ ultra sound; ^4^ uterine artery embolization; ^5^ placenta accreta spectrum; ^6^ differential diagnosis; ^7^ magnetic resonance imaging.

## Data Availability

The authors confirm that the data supporting the findings of this study are available within the article.

## Supplementary Materials

The following supporting information can be downloaded at: https://www.mdpi.com/article/10.3390/medicina61111915/s1, Table S1: PRISMA guidelines for systematic reviews; Table S2: Joanna Briggs Institute (JBI) Critical Appraisal Checklist for Case Reports.
