# Supplementary material for: Uterine Ectopic Pregnancies and Live Births: Systematic Review of the Literature and Concepts Underlying Favorable Outcomes
Source: Medicina (Kaunas). 2025 Oct 25;61(11):1915. doi: 10.3390/medicina61111915 (PMC12653997; doi:10.3390/medicina61111915)
Supplement: Supplementary file 1 [file medicina-61-01915-s001.zip › JBI Live birth - Foglio1.pdf]

Title	Author, Year	D1	D2	D3	D4	D5	D6	D7	D8
Cervical ectopic pregnancy – the first case of live birth and uterus-conserving management	Angela Köninger et al., 2023	YES	YES	YES	YES	YES	YES	YES	YES
Cervico-isthmic pregnancy: an extremely rare case diagnosed during labour	Spryros A. Mesogitis et al, 2001	YES	YES	YES	YES	YES	YES	YES	YES
Cervical Pregnancy: Case Reports and Current Concepts in Diagnosis and Treatment	H. M. H. Hofmann et al. 1987	YES	YES	YES	YES	YES	YES	YES	YES
Cervico-isthmic pregnancy ending with the delivery of a live-born infant in late second trimester	I. Cohen et al 1985	YES	YES	YES	YES	YES	YES	YES	YES
Diagnosis pitfall of interstitial pregnancy: a case report of a term pregnancy with abnormal placentation	Fatemeh Sadat Najib et al., 2021	YES	YES	YES	YES	YES	YES	YES	YES
Interstitial Pregnancy in the Third Trimester with Severe Preeclampsia: A Case Report and Literature Review	Shiho Nagayama et al. 2020	YES	YES	YES	YES	YES	YES	YES	YES
An evident asymmetrical uterus during cesarean delivery	Aiko Kakigano et al. 2018	UNCREAR	UNCLEAR	YES	YES	YES	YES	YES	YES
Interstitial Pregnancy Resulting in a Viable Infant Coexistent with Massive Perivillous Fibrin Deposition: A Case Report and Literature Review	Yusuke Tanaka et al. 2014	YES	YES	YES	YES	YES	YES	YES	YES
A True Cornual (Interstitial) Pregnancy Resulting in a Viable Fetus	Alexandria J. Hill et al. 2013	YES	YES	YES	YES	YES	YES	YES	YES
Antenatal diagnosis of a third trimester interstitial pregnancy: A case report	Anibal Scarella et al. 2012	UNCLEAR	YES	YES	YES	YES	UNCLEAR	YES	YES
Term interstitial pregnancy with uterine conservation	P.H. Ng et al. 2007	UNCLEAR	UNCLEAR	UNCLEAR	UNCLEAR	YES	YES	UNCLEAR	YES
Survival of cornual (interstitial) pregnancy	T.O. Idama et al. 1998	YES	YES	YES	YES	YES	YES	YES	YES
Live infant salvagedfrom a ruptured cornual (interstitial) pregnancyat 33-weeksgestation	A.H.N. Ugwumadu Et al 1997	YES	YES	YES	YES	YES	YES	YES	YES
Intramural ectopic pregnancy with live birth at 37 weeks of gestation	Laurent Petit et al. 2012	UNCLEAR	YES	YES	YES	YES	UNCLEAR	YES	YES
